# Supplementary figures and images for: Two forms of Opa1 cooperate to complete fusion of the mitochondrial inner-membrane
Source: eLife. 2020 Jan 10;9:e50973. doi: 10.7554/eLife.50973 (PMC7299343; doi:10.7554/eLife.50973)

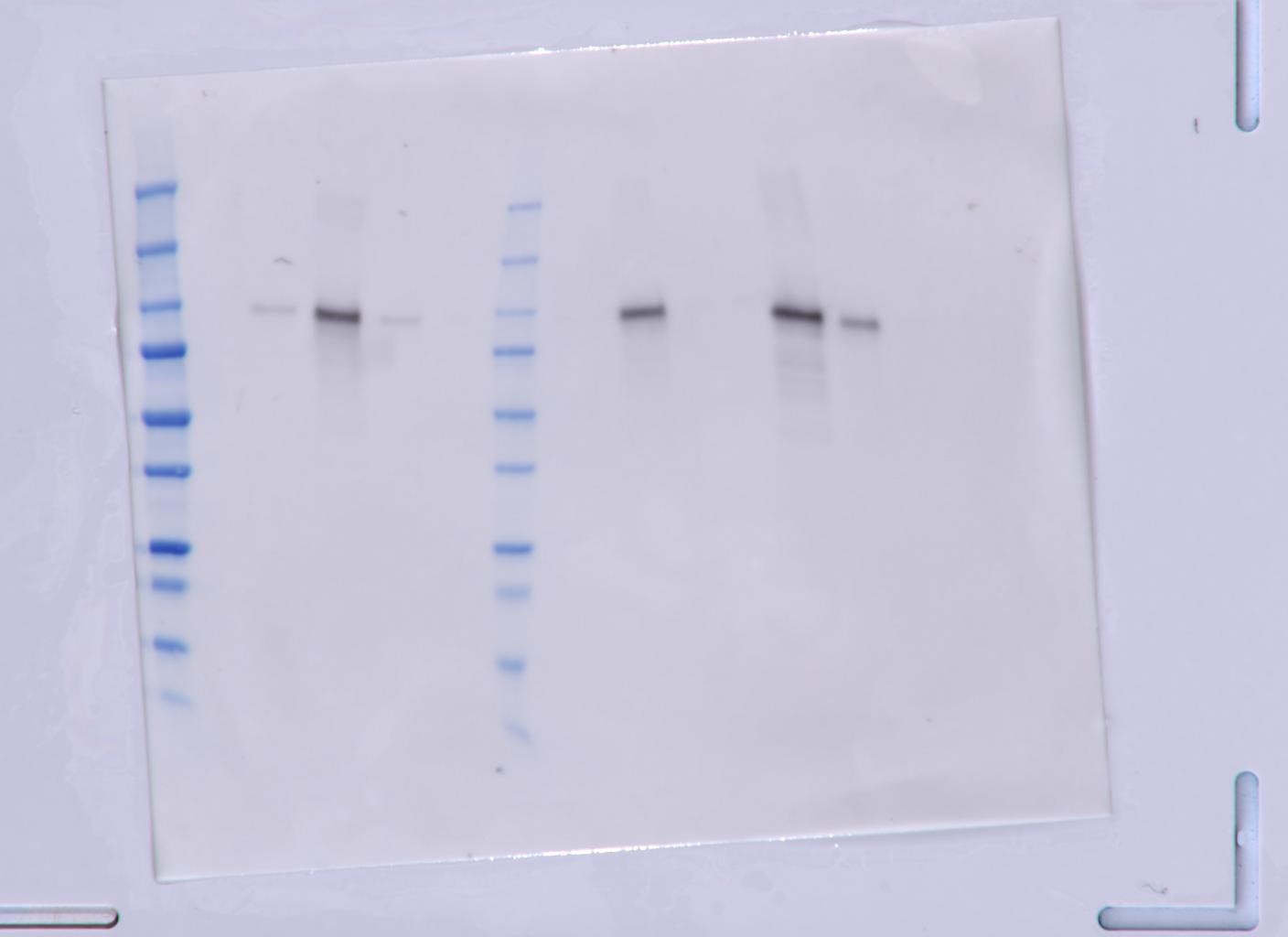

Supplement: Figure 2—figure supplement 2—source data 1. [file elife-50973-fig2-figsupp2-data1.zip › Figure 2-fig sup 2-source data1/Figure 2-fig sup 2 G (l) Figure 2-fig sup 2 I (r) .jpg]

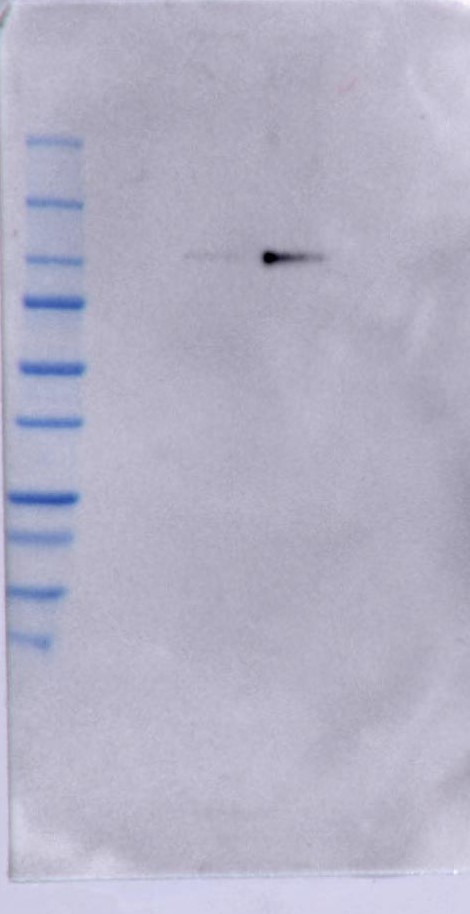

Supplement: Figure 2—figure supplement 2—source data 1. [file elife-50973-fig2-figsupp2-data1.zip › Figure 2-fig sup 2-source data1/Figure 2-fig sup 2 D.jpg]

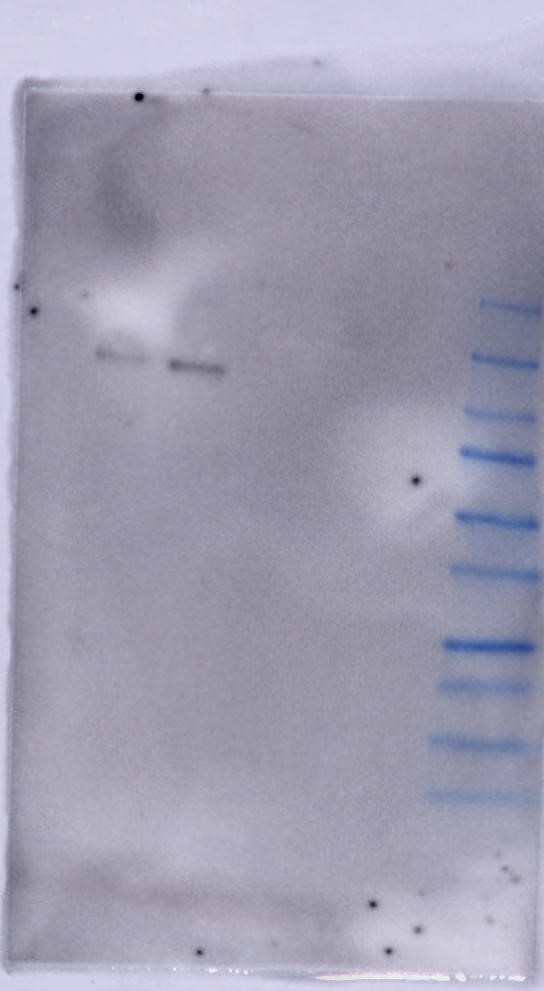

Supplement: Figure 2—figure supplement 2—source data 1. [file elife-50973-fig2-figsupp2-data1.zip › Figure 2-fig sup 2-source data1/Figure 2-fig sup 2 E.jpg]

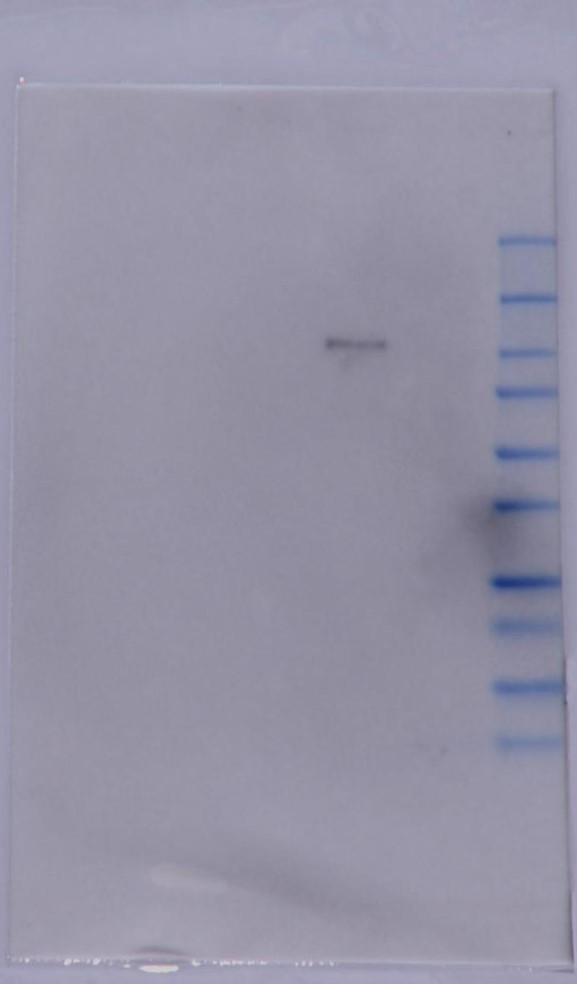

Supplement: Figure 2—figure supplement 2—source data 1. [file elife-50973-fig2-figsupp2-data1.zip › Figure 2-fig sup 2-source data1/Figure 2-fig sup 2 F.jpg]

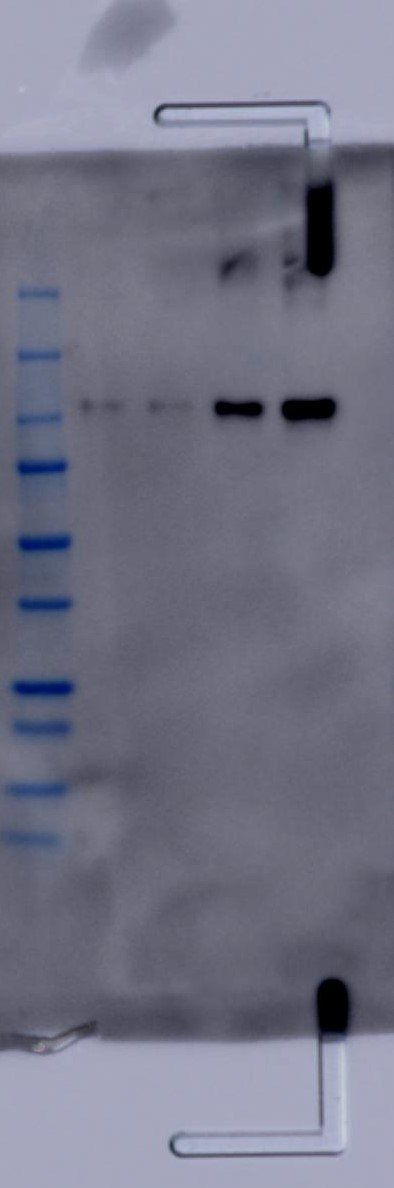

Supplement: Figure 2—figure supplement 2—source data 1. [file elife-50973-fig2-figsupp2-data1.zip › Figure 2-fig sup 2-source data1/Figure 2-fig sup 2 B.jpg]

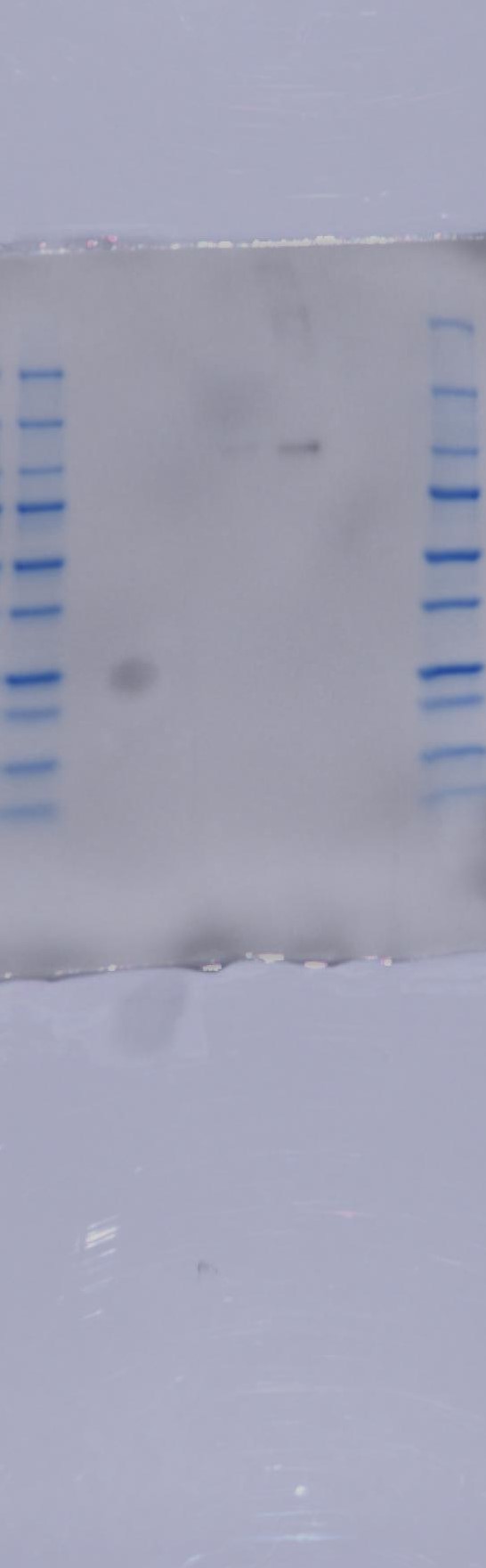

Supplement: Figure 2—figure supplement 2—source data 1. [file elife-50973-fig2-figsupp2-data1.zip › Figure 2-fig sup 2-source data1/Figure 2-fig sup 2 C.jpg]

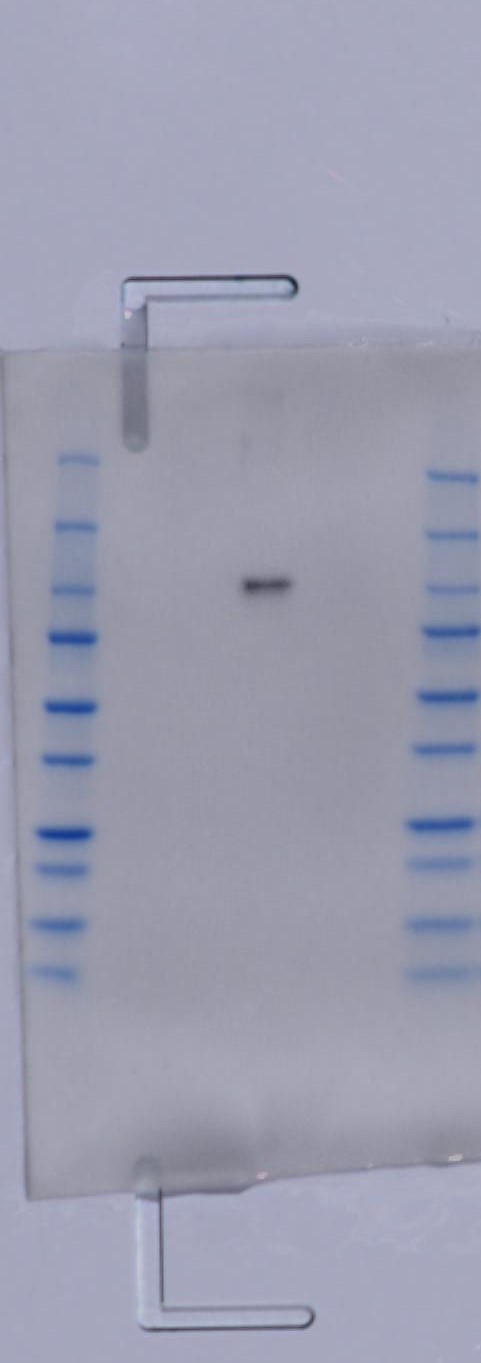

Supplement: Figure 2—figure supplement 2—source data 1. [file elife-50973-fig2-figsupp2-data1.zip › Figure 2-fig sup 2-source data1/Figure 2-fig sup 2 A.jpg]

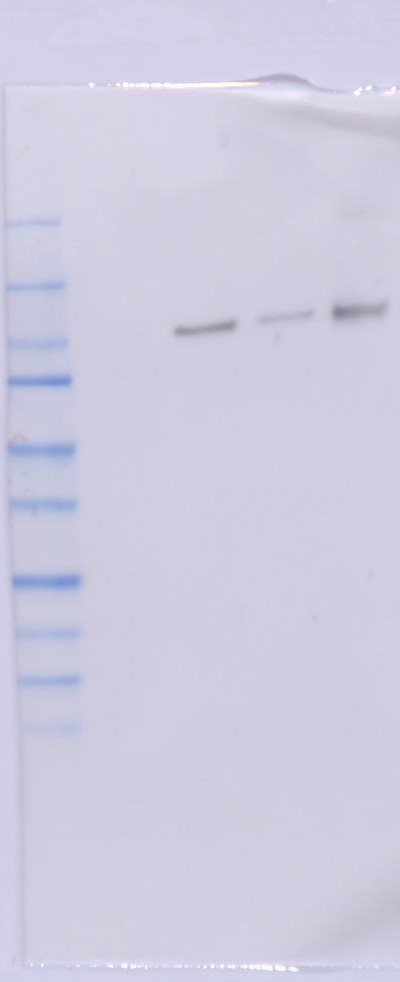

Supplement: Figure 2—figure supplement 2—source data 1. [file elife-50973-fig2-figsupp2-data1.zip › Figure 2-fig sup 2-source data1/Figure 2-fig sup 2 L.jpg]

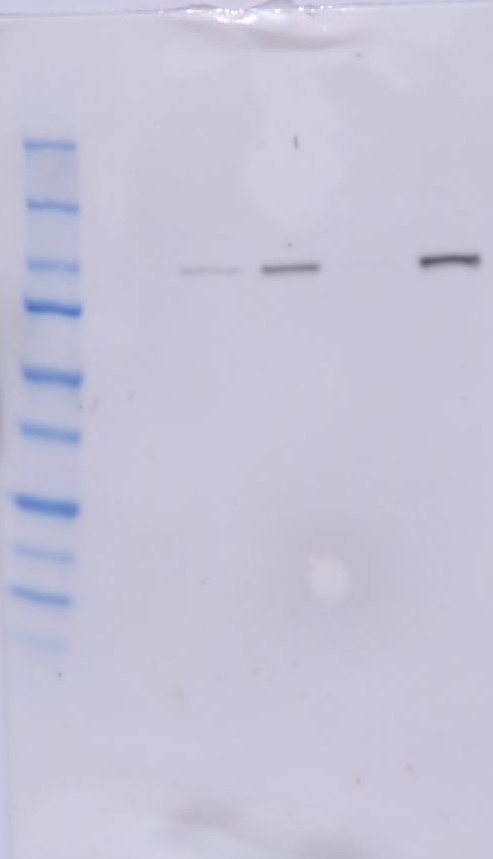

Supplement: Figure 2—figure supplement 2—source data 1. [file elife-50973-fig2-figsupp2-data1.zip › Figure 2-fig sup 2-source data1/Figure 2-fig sup 2 K.jpg]

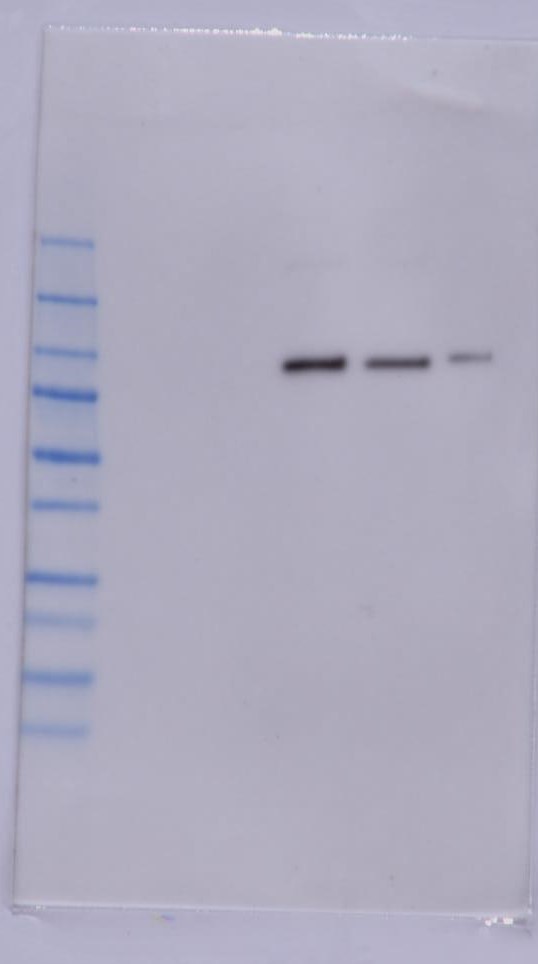

Supplement: Figure 2—figure supplement 2—source data 1. [file elife-50973-fig2-figsupp2-data1.zip › Figure 2-fig sup 2-source data1/Figure 2-fig sup 2 J.jpg]

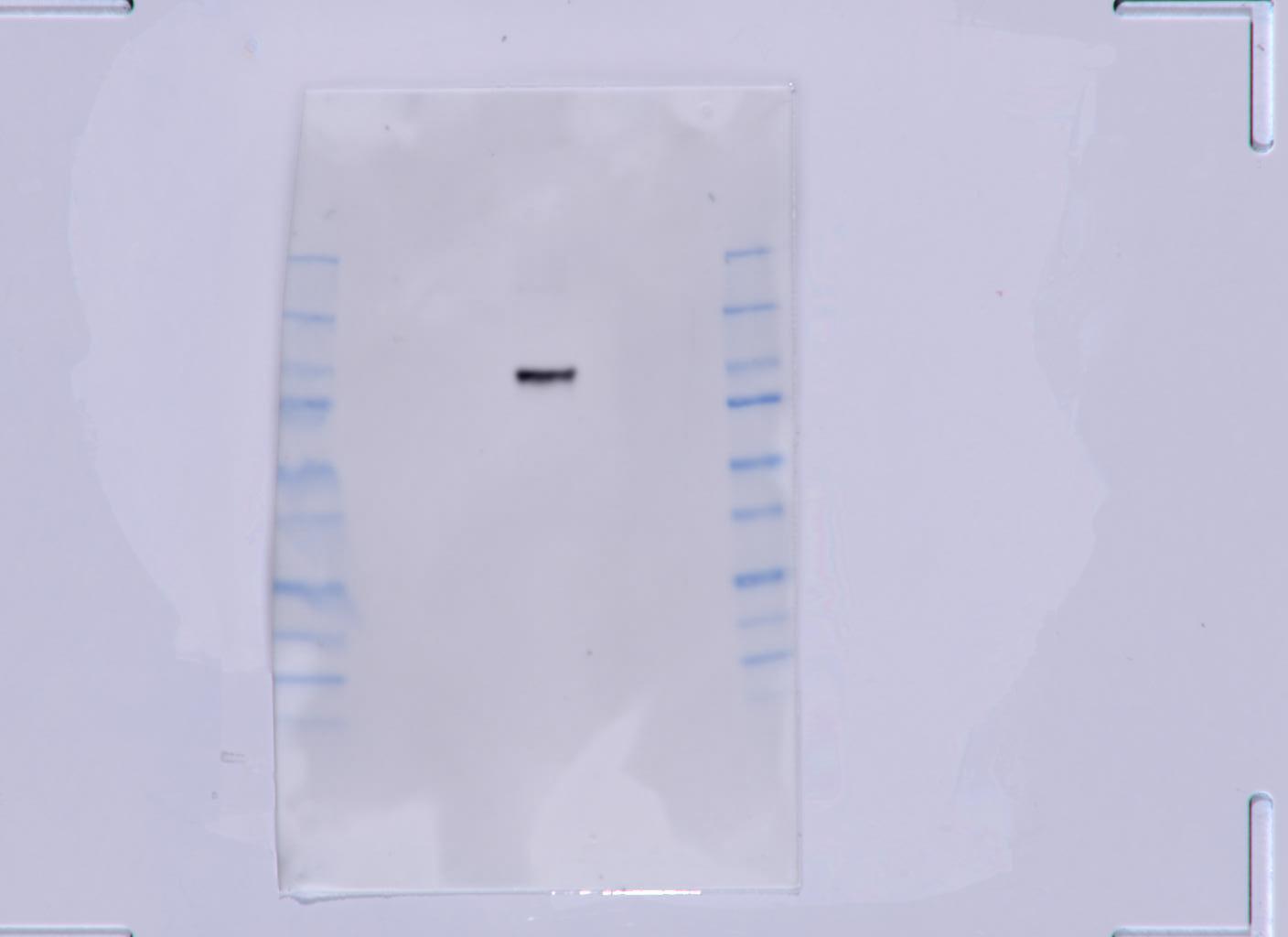

Supplement: Figure 2—figure supplement 2—source data 1. [file elife-50973-fig2-figsupp2-data1.zip › Figure 2-fig sup 2-source data1/Figure 2-fig sup 2 H.jpg]
